# Abnormal oscillatory brain dynamics in schizophrenia: a sign of deviant communication in neural network?

**DOI:** 10.1186/1471-244X-7-44

**Published:** 2007-08-30

**Authors:** Brigitte S Rockstroh, Christian Wienbruch, William J Ray, Thomas Elbert

**Affiliations:** 1 Department of Psychology, University of Konstanz, PO Box D23 D-78457, Konstanz, Germany; 2 Department of Psychology, Pennsylvania State University, University Park, PA, USA

## Abstract

**Background:**

Slow waves in the delta (0.5–4 Hz) frequency range are indications of normal activity in sleep. In neurological disorders, focal electric and magnetic slow wave activity is generated in the vicinity of structural brain lesions. Initial studies, including our own, suggest that the distribution of the focal concentration of generators of slow waves (dipole density in the delta frequency band) also distinguishes patients with psychiatric disorders such as schizophrenia, affective disorders, and posttraumatic stress disorder.

**Methods:**

The present study examined the distribution of focal slow wave activity (ASWA: abnormal slow wave activity) in116 healthy subjects, 76 inpatients with schizophrenic or schizoaffective diagnoses and 42 inpatients with affective (ICD-10: F3) or neurotic/reactive (F4) diagnoses using a newly refined measure of dipole density. Based on 5-min resting magnetoencephalogram (MEG), sources of activity in the 1–4 Hz frequency band were determined by equivalent dipole fitting in anatomically defined cortical regions.

**Results:**

Compared to healthy subjects the schizophrenia sample was characterized by significantly more intense slow wave activity, with maxima in frontal and central areas. In contrast, affective disorder patients exhibited less slow wave generators mainly in frontal and central regions when compared to healthy subjects and schizophrenia patients. In both samples, frontal ASWA were related to affective symptoms.

**Conclusion:**

In schizophrenic patients, the regions of ASWA correspond to those identified for gray matter loss. This suggests that ASWA might be evaluated as a measure of altered neuronal network architecture and communication, which may mediate psychopathological signs.

## Background

Slow wave electrocortical activity composed of large amplitude and low frequency activity in the delta (0.5–4 Hz) or theta (4–7 Hz) frequency bands can be seen in normal sleep stages; this has been mainly reported for electroencephalogram (EEG) but also for magnetoencephalogram (MEG) measurements [1]. Although mainly associated with brain quiescence and the closing of thalamic gates for external input, such activity has also been related to unexpected high levels of spontaneous neuronal activity, which may serve important cognitive processes such as memory consolidation [2,3]. This raises the question of how to understand slow wave activity in waking states. Traditionally, delta activity in the waking state has mainly been of interest to those studying neurological disorders. Slow wave rhythms are found in a variety of developmental and degenerative disorders, in toxic and metabolic encephalopathy and in other neurological conditions. Focally concentrated slow waves appear in the vicinity of a structural lesion such as cerebral infarct, contusion, local infection, tumor, epileptic foci and subdural hematoma [4-16]. Abnormal slow wave activity (ASWA) has been attributed to a 'dysfunctional state' of the generating neuronal tissue [17], due to changes in metabolism and blood flow consequent upon insult [11,12,18] or due to 'deafferentation' of neural networks that have been cut off from major input sources.

Generators of slow wave activity in cortical and subcortical structures were initially determined from intracranial electrodes. More recently, magnetic source imaging (MSI) has been used to identify cortical generators through dipole density measurement from the MEG [7,8,12,13,19,20]. Using this procedure, we confirmed left-hemispheric slow wave clusters in aphasic patients suffering from ischemic or hemorrhagic lesions affecting the left hemisphere [21].

Abnormalities in the EEG or MEG power spectra have also been reported in psychiatric disorders. For instance, augmented activity in the lower EEG- or MEG-frequency bands which can be either diffuse or focused over distinct brain regions has been reported for a variety of psychiatric disorders such as schizophrenia [22-27], dementia [20,28,29], and posttraumatic stress disorder (PTSD; [[30], and: Kolassa IT, Wienbruch C, Neuner F, Schauer M, Ruf M, Odenwald M, Elbert T: Imaging the trauma: altered cortical dynamics after repeated traumatic stress, submitted]. It has also been observed in symptoms related to deafferentation such as tinnitus [31], which is linked to hearing loss. Overall, such spectral abnormalities seem to be related to brain reorganization in response to lesion and deafferentation rather than to a particular disease. Llinas and coworkers [32] concluded from the magnification of MEG-slow waves in various disorders (e.g., tinnitus, obsessive compulsive disorder, depression) that the surface phenomenon reflected subcortical dysfunction or thalamo-cortical dysrhythmia. Using magnetic source imaging and dipole density measurement, researchers have also found specific distributions of abnormality in network dynamics of schizophrenic patients [19,33-36].

The present study used spontaneous MEG as bases for source analyses, which has some advantages for this purpose in comparison to EEG. One advantage is that MEG by its nature separates focally generated slow waves from those with widespread sources [37]. Another advantage is that MEG is better able than EEG to identify focal sources located in sulci which comprise two-thirds of the cortex. Thus, the same brain processes are pictured differently by MEG and EEG, and combined measurements of both MEG and EEG quantitatively illustrate this point [38].

The purpose of the present study is to extend our previous results that were obtained from smaller samples [19,35] by employing a more specific method of slow wave mapping with a sample of 116 healthy subjects, a sample of 42 individuals with affective (F3) or neurotic/reactive (F4) disorders and a sample of 76 patients with diagnoses from the schizophrenia spectrum. Given the ability of focal abnormal slow wave mapping to disclose dysfunctional or reorganized brain areas in neurology [21], we asked whether the distribution of ASWA or its focal concentration might index altered brain areas even in the absence of structurally obvious lesions, thereby offering a signature of potentially dysfunctional neuronal network architecture and communication in psychiatric disorders as additional diagnostic information. Given a potentially clinical relevance, we designed recording and analyses brief and economic, and we did not pre-select specific subgroups but rather evaluated potential impact of medication and substance abuse in post-hoc analyses.

Whereas a number of studies have verified the significance of abnormal slow waves in neurological disorders, its significance in psychiatric patients still has to be substantiated. Therefore, the present study sought to determine if abnormal slow wave activity would also be present in individuals with a variety of psychopathologies. From previous studies including our own, we expected significant differences of ASWA (intensity and distribution) in psychiatric patients compared to healthy subjects. Further, we expected more abnormal slow wave activity in schizophrenia patients than in healthy subjects. Additionally, exploratory analysis with relevant clinical variables will help to inform future research regarding the functional significance of ASWA.

## Methods

### Design

Five-minute recordings of spontaneous MEG were obtained from 116 healthy subjects, 76 individuals with schizophrenia and 42 individuals with affective disorders. No task or intervention was induced.

### Participants

The *schizophrenia *sample consisted of 76 inpatients (62 males, 14 females; 29.12 ± 8.0 years of age (mean ± SD) diagnosed and treated for schizophrenia or schizoaffective disorder from a university inpatient research unit at the local Center for Psychiatry. Male patients were non-significantly younger (28.53 ± 7.9 years) than females (31.71 ± 7.7 years; t(74) = 1.36, p = .18). Diagnosed by experienced senior psychiatrists using ICD-10 criteria, the majority of patients (N = 61, 49 male) received F20 diagnoses, the remaining subjects receiving diagnoses of schizoaffective disorder (F25, N = 9, 7 male) or F23 category (N = 6, 6 male). Duration of illness varied between 0 (first admission) and 22 years around a mean of 5.78 ± 5.8 years, and did not differ between male (5.40 ± 5.9) and female patients (7.5 ± 5.3; t(63) = 1.13, p = .26). Mean age at first admission varied around a mean of 23.22 ± 5.4 years, with (non-significantly) earlier age of onset in male (22.77 ± 5.3 years) than in female patients (25.17 ± 6.0 years, t(63) = 1.39, p = .17). Duration of illness was related to age (r = .71, p = .0001), but not to the age of first admission (r = -.07). As co-diagnoses, 29 patients (all male) met the criteria of cannabis abuse (F12.1) or dependency (12.2), and 5 patients received the co-diagnoses of alcohol abuse (F19.1). Among the 40 patients without drug abuse history, 29 were male and 11 female. The symptom profile was characterized by PANSS (Positive and Negative Symptom Scale) [39] and BPRS (Brief Psychiatric Rating Scale) [40] (PANSS-P: 14.13 ± 5.0 (range 7–26); PANSS-N: 21.26 ± 6.3 (7–33); PANSS-G: 35.26 ± 7.7 (21–62); BPRS (available from 44 subjects): 42.67 ± 10.7 (25–79). At the time of the investigation only 7 patients were not under neuroleptic medication. The other patients received traditional (N = 14), atypical neuroleptics (N = 41) or a combination of both (N = 14). Eight patients were left-handed as verified by a modified version of the Edinburgh Handedness Questionnaire [41]. Since handedness may affect the topography of brain activity, in particular hemispheric asymmetry, analyses were repeated without the left-handers. Given that the results did not differ, we concluded that handedness did not have a crucial impact on the present dependent variables and report results for the entire sample of 76 patients.

The group of inpatients diagnosed and treated for *affective/neurotic disorders *(diagnostic categories below) comprised 42 (13 males, mean age 45.1 ± 8.5 years) subjects. Data from 20 patients from an earlier study [19] were re-analyzed with the new method, and 22 patients were additionally recruited for the present study. Eighteen patients were diagnosed as suffering from severe major depressive episode (ICD-10: F32), sixteen from recurrent depressive episodes (F33), and eight from neurotic, reactive and somatoform disorders (F4). In terms of medication, thirty-six patients received antidepressive medication including tricyclic antidepressants (TCA, N = 13), selective serotonin reuptake inhibitors (SSRI, N = 7), a combination of both (N = 5) or a combination with neuroleptics or mood stabilizers (N = 11). The severity of depressive symptoms at the time of the investigation was assessed by the Beck Depression Inventory (BDI, mean of 25.1 ± 9.91, range 1–49). In addition, the BPRS was available for 22 patients (mean ± SD 49.73 ± 10.9, range 32–77). All subjects in the depressive sample were right-handed as verified by the Edinburgh Handedness Questionnaire.

The sample of *healthy subjects *comprised 116 individuals (59 males, mean age 28.95 ± 10.16 years), who had never been treated for a neurological or psychiatric disorder and were not under medication (other than vitamins or contraceptives). All subjects were right-handed as verified by the Edinburgh Handedness Questionnaire. In all of our studies, healthy controls were matched in terms of age and gender to individuals with the psychiatric group investigated in the specific study. This resulted in a combined group of healthy subjects varying in age in relation to the psychopathology groups. The overall healthy subjects group was comparable to the schizophrenia sample with respect to age (t<1), whereas patients with affective diagnoses were significantly older than healthy subjects (t(156) = 9.21, p = .0001) and schizophrenic patients (t(116) = 10.20, p = .0001).

### Data acquisition and analysis

Using a 148-channel whole-head neuromagnetometer (MAGNES™ 2500 WH, 4D Neuroimaging, San Diego, USA) the MEG was measured during a 5-minute resting period using 678.17 Hz sampling rate and real bandpass filter of 0.1–200 Hz. Recordings were obtained in a lying position. Subjects were asked to relax but stay awake and not engage in any specific mental activity. They were further asked to fixate a mark on the ceiling of the magnetically shielded room throughout the recording in order to avoid eye- and head-movement. A video camera installed inside the chamber allowed monitoring the subject's behavior and compliance at any time throughout the measurement. The subject's index points and head shape were digitized with a Polhemus 3Space^® ^Fasttrack prior to each measurement. In specific the nasion point, an anatomical landmark, and the left and right ear canal points served as index points and were used to define a right handed coordinate system, called headframe coordinate system. The x-axis points to the front, the y-axis to the left and the z-axis to the top of the head. The headshape information is used in the standard analysis software for localization of activity sources (4D Neuroimaging – WHS 1.2.6) by fitting a local sphere to the head shape underneath the selected channel groups. The subject's head position relative to the pickup coils of the sensor was estimated before and after each measurement.

ASWA generators were identified in a semi-automated procedure. Following noise reduction, data were screened for artifacts (e.g., eye blinks, muscle activity) by visual inspection. Data were then reduced by a factor of 16 (anti-alias filters are applied automatically in the same processing step; new sampling rate of 42.4 Hz) and digitally filtered for the delta (1.5–4.0 Hz) frequency band using a digital band pass filter (Butterworth filter). Single equivalent current dipoles (ECD) were fitted for each time point in the selected artifact free segments; the selection of specific dipole solutions had to meet the criteria Goodness of Fit (GoF > 0.90, to ensure the statistical significance of the source model) and Dipole moment (Q 10 – 100 nAm, which may require that at least 0.1 – 1 cm^2 ^of cortex is activated). Dipoles with an inferior-superior coordinate (z) below 0.0 cm and dipoles within a sphere with a radius of 3 cm around the origin were excluded. Dipole density was estimated within a volume defined as a cube divided into voxel with a size of 2 × 2 × 2 cm^3^. The cube is determined from an ACPC (anterior commissural (AC) – posterior commissural (PC)) based coordinate system, which, in turn, results from the individual headframe coordinate system, defined by the nasion, the left and the right ear channel). Each source volume (cube) comprises 1331 voxel. Each equivalent dipole is assigned to one voxel of the source volume, and the number of dipoles within a voxel is counted. As artifact-free time periods vary between subjects the dipole density was normalized by the subject's individual number of artifact free time points.

The normalized dipole density was calculated for each subject within each voxel of the source volume; then the logarithm of the normalized dipole density was calculated in order to obtain a normal distribution across subjects. For group comparison and visualization the distribution was z-transformed using mean and standard deviation from the healthy subjects group. This score of ASWA, for the deviation from normal was labeled Z. For the analysis of the ASWA distribution, dipole density was determined within anatomically defined brain areas, which were specified for temporal, frontal, central and parietal areas of the brain within each hemisphere following the classification of the anatomical atlas provided with MRICRO's AAL [42]. Within these 8 regions dipole density was estimated as the average of the voxel-based dipole density (that is, Z). The entire procedure is described in detail elsewhere [43].

For the dependent variable Z, group differences and topographical characteristics were evaluated by repeated-measures analyses of variance (ANOVA) including the between subjects variables Group (comparing the two patient groups and the healthy subjects group), Gender (comparing male and female subjects within each sample), Hemisphere (comparing the 4 left- and the 4 right-hemispheric regions) and Region (comparing the anatomically defined frontal, temporal, central, and parietal cortical areas). Normal distribution of the data of all three samples was evaluated in each of the eight regions using the Shapiro-Wilks test. Separate ANOVA and correlation analyses served to explore the effects of clinical variables (like medication, drug abuse, and duration of illness) on the ASWA distribution within the patient groups.

## Results

The interaction Group × Region (F(6,687) = 12.30, p < .0001), and main effects Group (F(2,229) = 18.13, p < .0001) and Region (F(3,687) = 4.17, p < .001) suggest significant and region-specific differences between groups. These three effects remained statistically significant, if age was included as a covariate or if the age-range was restricted to 30–50 years within each group.

**Figure 1:**
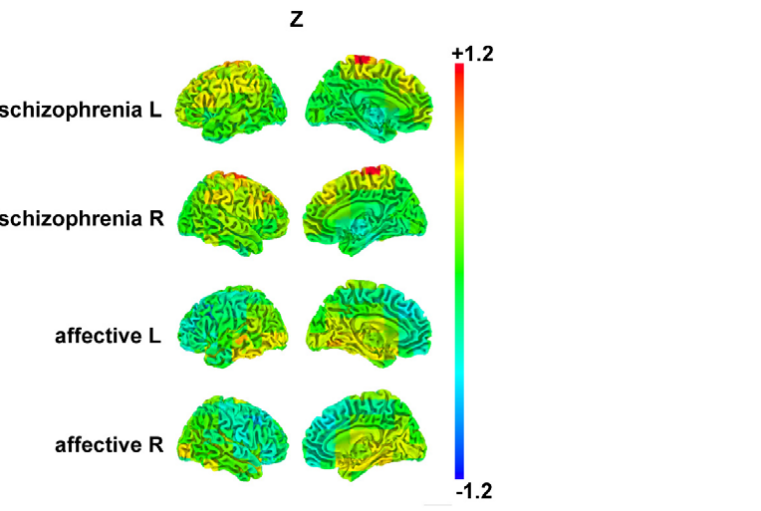
Projection of Z-scores (see method section for description of this measure) for patients with schizophrenia (upper two rows) and with affective or neurotic/reactive diagnoses (lower two rows) onto outer surface (left column) and inner view (second left column) of the brain hemispheres. The letters 'L' and 'R' behind the diagnostic groups indicate the left hemispheric and the right hemispheric views. Z-scores depict ASWA relative to the group of 116 healthy subjects with colors indicating the range of deviation from the healthy subject group; they vary between large deviation (red color = Z-scores > 0.4) to reverse deviation (lower ASWA relative to the healthy subject group; blue color = Z-scores > -0.4); small to negligible deviation from normal is depicted by green color. The segmented brain was calculated using the software package Caret (Van Essen DC, Drury HA, Dickson J, Harwell J, Hanlon D, Anderson CH (2001) An integrated software suite for surface-based analyses of cerebral cortex. J Am Med Inform Assoc, 8: 443–59), overlays of ASWA and the voxel based t-test results are based on AFNI/SUMA (Cox RW (1996) AFNI: software for analysis and visualization of functional magnetic resonance neuroimages. Comput Biomed Res, 29, 162–73).

**Figure 2:**
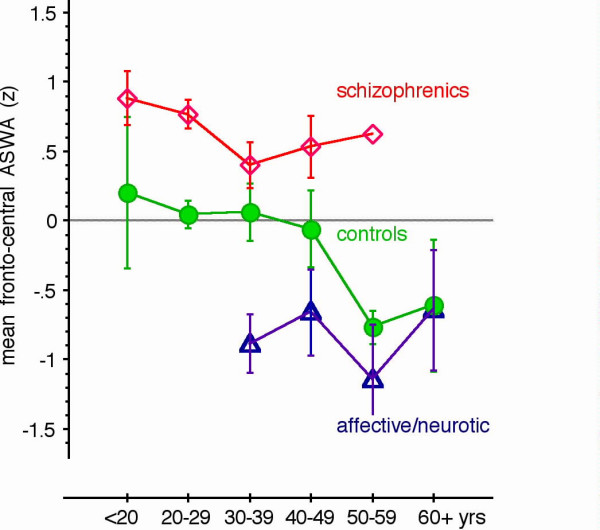
Mean fronto-central ASWA (Z-scores averaged across the left- and right-frontal and the left- and right central regions, ordinate) are plotted separately for the three groups (filled circles: controls, squares: schizophrenia patients, triangles: patients with affective or neurotic/reactive diagnoses) and for age categories (abscissa: <20 yrs: n = 4 controls, 5 schizophrenics, 0 affective/neurotic patients 17–19 years of age; 20–29: n = 74 controls, 34 schizophrenics, 0 affective/neurotic patients between 20 and 29 years of age; 30–39: n = 26 controls, 28 schizophrenics, 10 affective/neurotic patients between 30 and 39 years; 40–49: n = 4 controls, 8 schizophrenics, 15 affective/neurotic patients between 40 and 49 years; 50–59: n = 3 controls, 1 schizophrenic, 13 affective/neurotic patients between 50 and 59 years; 60+: n = 5 controls, 0 schizophrenic, and 3 affective/neurotic patients 60 years and older). Symbols indicate mean values, bars standard deviation.

Figure 1 illustrates areas of slow wave activity in patients with schizophrenia and affective/neurotic disorders, which differ from the topography in the healthy subjects group. Enhanced ASWA was found in schizophrenic patients (for the subset of data including only healthy subjects and schizophrenia patients: Group, F(1,190) = 19.19, p = .0001). The abnormality was accentuated in central and frontal areas (Group × Region, F(3,570) = 4.84, p = .0025). In contrast, the inpatients with affective diagnoses exhibited less ASWA than healthy subjects in frontal and central regions, whereas ASWA did not differ significantly from healthy individuals in posterior and temporal regions. Figure 2 illustrates that the fronto-central group differences emerge for the entire age range tested.

Post-hoc planned comparisons confirmed significant differences between healthy subjects and schizophrenic patients, healthy subjects and affective/neurotic disorder patients, and schizophrenic and affective/neurotic disorder patients for frontal (p < .0001) and central (p < .0001) regions, but not for parieto-occipital (p > .1) and temporal regions (where only schizophrenic patients tended to show larger ASWA than healthy subjects, t(190) = 1.86, p = .065).

Within the *schizophrenia *sample ASWA distribution did not differ between gender. Patients treated with traditional neuroleptics (N = 14) displayed the least ASWA whereas unmedicated patients (N = 7) showed the most pronounced ASWA. In patients under atypical medication or a combination of traditional and atypical was more similar to unmedicated than to traditionally medicated patients (medication group, F(3,72) = 2.73, p = .05); the distribution of ASWA was not affected by medication (Medication group × Region, F<1). Whereas general evaluation of the symptom state by PANSS and BPRS sum scores was not significantly related to ASWA or its distribution, the average fronto-central ASWA was correlated with the BPRS items reflecting affective symptoms: Higher BPRS-depression scores were related to less fronto-central ASWA (r = -.30, p = .06), but higher BPRS-affective flattening, if at all, tended to be related to more frontal ASWA (r = .28, p = .09). In particular those subjects with more pronounced affective flattening and less pronounced depression (defined as the difference between the two scores) displayed more fronto-central ASWA (r = .46, p < .01). Multiple regression analysis confirmed that average fronto-central ASWA could be significantly predicted (r^2 ^= .56, F = 5.15, p = .005) by BPRS affective flattening, depression (negative load) and PANSS-P (but not by age, duration or age of hospitalization or other symptom scales).

There was a complex relationship between ASWA distribution, demographic and clinical variables: Age varied with ASWA and affected BPRS-depression in male patients (r = +41, p = .02), but did not contribute significantly to the relationship between fronto-central ASWA and symptoms in regression analysis. Symptom severity (assessed by the BPRS) varied with medication, being higher in unmedicated patients than in patients treated with traditional (t(10) = 2.32, p = .043) and atypical neuroleptics (t(30) = 3.82, p = .0006), while BPRS-depression score was lower under atypical medication than in non-mediated patients (t(26) = 2.66, p = .013). But whereas groups markedly differed in symptoms, there was no significant difference in the distribution of ASWA between subjects receiving atypical neuroleptics and unmedicated patients. Cannabis-abuse was not related to symptom scores or medication, although cannabis-abusers were younger than non-users (t(72) = 4.09, p = .0001).

Within the sample of patients with *affective or neurotic diagnoses*, multiple regressional analysis disclosed that BPRS-depression could be predicted from the mean frontal (average of left- and right-frontal region) and the mean parieto-occipital ASWA (F = 3.59, p = .047); this relationship was mainly explained by frontal ASWA, that is, those patients who were rated as more depressed by psychiatrists, exhibited less frontal slow wave activity (R = -.48, t = -2.56, p = .02), but a tendency towards more parieto-occipital ASWA (R = .27 p = .07).

## Discussion

The present study builds upon previous studies showing that the distribution of generators of slow wave activity in the delta (0.5–4 Hz) frequency range distinguishes individuals suffering from psychiatric disorders from healthy individuals. Overall, we found that both individuals with schizophrenia and those with affective or neurotic/reactive diagnoses differ from healthy controls. In particular, schizophrenic patients show more abnormal slow wave activity (ASWA) than both a patient group with affective/neurotic diagnoses and a group of healthy subjects. Conversely, the affective/neurotic disorder group showed less slow wave activity than the other two groups in frontal and central areas. Less frontal activity was also seen in schizophrenia patients with higher BPRS-scores of depression. Thus, our results demonstrate the ability of ASWA to distinguish between psychopathological groups and controls. This raises two questions: (a) what factors may be driving abnormal slow wave activity and (b) can the measurement add clinically useful information?

Schizophrenic patients in our present study are characterized by extended and excessive slow wave activity, which adds to earlier findings from our [19,35,36] and other groups [33,34,44,45]. An important finding of the present study is the correspondence of abnormal slow wave activity in those areas that previous studies have associated with gray matter loss in schizophrenia [e.g. [46,47]]. In particular we found the prominence of abnormal slow wave activity to be in the central area of the cortex comprising anatomical areas from precentral and supplementary motor areas to superior and inferior parietal lobes and the middle part of the cingulate gyrus, and in frontal regions comprising orbitofrontal, medial and superior frontal areas with the latter corresponding to dorsolateral prefrontal cortex and the anterior part of the cingulum. Centro-parietal) ASWA was even more pronounced in younger patients with schizophrenia whereas the variation of age with anterior (centro-frontal) ASWA only approached significance. Although the present data do not allow specific conclusions in this respect, they may be hypothetically related to reports of a more posterior start of gray matter loss in adolescents with early onset schizophrenia and with later progression to frontal (including orbitofrontal and dorsolateral prefrontal cortex [48-50] and temporal regions [51]). A widespread distribution of ASWA could be discussed within the framework of a neurodevelopmental model of schizophrenia [52] as one of the "downstream or tangential manifestations of the core neurobiological phenotype, viz. the genetically influenced molecular disruption of neural circuits" (p. 41). One intriguing possibility is that morphological changes like loss of gray matter provide one basis or substrate for functional changes in neuronal network architecture and communication. As to what extent this could be related to weakened connectivity, which has been hypothesized for schizophrenic disorders [53], remains to be substantiated by combining ASWA mapping with measures of connectivity in future studies.

It should be noted that the present results of extended ASWA in schizophrenic patients replicate our previous findings, although they did not substantiate a left-hemispheric and temporal focus of ASWA as described in [19,35]. These differences may be attributed to three differences between the present study and our previous work. First, in the current study we followed a more precise anatomical atlas [42] for the definition of regions of topographical analysis, whereas previous work measured activity simply in eight cubes of equal size. Thus, it may well be that the presently defined frontal and central regions included areas such as the cingulum or orbitofrontal cortex that had been attributed to the 'temporal' cubes in the former study. Second, the present analysis comprised of a more detailed determination of slow wave generators within voxels, which might have resulted on a more sensitive detection of ASWA. Third, the present study included a larger sample size, which could have influenced the results: The present group of 116 healthy subjects might represent 'normality' better than a control group of some 20 subjects. Likewise, the present sample of 76 schizophrenic patients might represent a larger and, hence, more realistic spectrum of clinical characteristics than the previous more strictly selected sample of some 20 male patients, all meeting a F20 diagnoses and other selection criteria.

In schizophrenic patients anterior ASWA varied with affective symptoms. This was best seen in terms of a lack of affective modulation rather than with depressive affect. Although this could be related to affective modulation in orbitofrontal regions, it does seem intriguing that dysfunctional neuronal network communication did not vary with more cognitive dysfunction – as was found in earlier studies [19] and also in relation to gray matter loss [51,54]. However, a complex and dynamic transition from causes of schizophrenia to the level of symptoms might not become manifest in a simple relationship between ASWA (measured under laboratory conditions) and those clinical measures that are used to characterize the schizophrenic state on the ward (like BPRS and PANSS).

In order to rule out alternative explanations for our results, we examined demographic and clinical variables in relation to ASWA. The gender effect on ASWA is a potential confound. However, results did not change, when gender is included as a between-subjects factor. In particular, there was no interaction with the factor for diagnostic groups. In addition, we probed the gender effect separately in the diagnostic groups: the 14 female schizophrenics displayed pronounced frontal and temporal ASWA similar to that of the 62 male patients. In fact, the frontal ASWA excess in female schizophrenia patients contrasts the "female" pattern of lower-than-average frontal ASWA, which was displayed by controls. In the affective/neurotic sample, the 13 male patients did not differ from the 28 female subjects with respect to lower-than-normal frontal and equal-to-normal central ASWA. Effects of medication on the human EEG/MEG always present a potential confound in the interpretation of data of any study, but are difficult to evaluate, as unmedicated patients may differ in most clinical studies in their symptom profile from medicated ones. Moreover, findings for the influence of medication may be two-fold: The drug may act directly on brain activity and/or indirectly via clinical improvement. Therefore it seems not surprising that results are inconsistent: as a slowing of EEG frequencies has been reported as a consequence of neuroleptic medication, but also 'normalizing' effects [27,55-57]. In the present study, there was only a suggestion that typical neuroleptics like haloperidol reduced ASWA more than atypical neuroleptics. Given that assignment to medication was not random, but followed clinical guidelines, more specific conclusions about the effect of medication on ASWA must remain speculative. In addition, clinical variables rarely exist independently. For example, symptom scores might vary with duration of illness, medication or cannabis abuse, and in their combination or interaction affect neuronal network communication.

The present study also found lower-than-normal slow wave activity in patients hospitalized for affective/neurotic disorders, mainly depression. Decreased EEG slow wave activity in depression has been reported before [58,59]. Our results disclosed a pattern of less slow wave activity, mainly in the anterior central regions and mainly in patients suffering from recurrent depressive episodes which might signal a more severe form of the disorder. From quantitative EEG (qEEG) analysis, Pozzi et al. [59] distinguished a posterior increase in delta power in depressed patients with dementia compared to a global decrease in delta power in depressives without dementia. Posterior dominance of ASWA has been demonstrated in patients with Alzheimer dementia [29], but a relationship between depression and dementia was not explored in the present sample. Indirect support for the pathological significance of reduced slow wave activity can be inferred from its (left-frontal) increase after successful electroconvulsive therapy [60]. Moreover, PET studies disclosed hypometabolism in the inferior frontal lobe [61,62] and prefrontal cortex [63] in depression. However, comparability to other findings does not allow more specific hypotheses about the functional meaning of the lower frontal ASWA in affective/neurotic disorders. As to what extent the lower-than-normal frontal ASWA characterizes affective psychopathology or the anterior-posterior gradient, that is, more pronounced ASWA in posterior regions, must to be clarified by future studies.

A final question is to what extent ASWA markers may assist in diagnosis and treatment. Historically, Lewine & Orrison [17] recommended the method and the measure for the mapping of dysfunctional brain tissue. Derived from neurological evidence, 'dysfunction' was described as a state of less information transfer within and across networks. These slow oscillations were thought to indicate less exchange of excitation as they have been related to hypometabolism and reduced blood flow [18]. De Jongh [8,9] suggested that ASWA results from damage done by structural lesion on surrounding white/gray matter rather than resulting from the lesion itself. The surrounding tissue may be considered in a state of disconnection or de-afferentation. The present paper along with previous ones demonstrates that ASWA may be seen in psychopathology. One possibility would therefore be to test this measure as an indicator for the progress in the damage of brain circuits. For example, patients with high but not low values might be tested if they respond positively to attempts to ameliorate dementia and to stimulate neurogenesis.

## Conclusion

The different patterns of ASWA in groups of patients with different psychiatric diagnoses suggest that mapping abnormal slow wave activity might allow for differentiating individuals with psychiatric diagnoses from normal controls and potentially also to differentiate different diagnostic groups. As a relatively new measure, ASWA at present must be considered a descriptive measure for which quite different explanations have been proposed. A crucial next step in this process of evaluation would be further clarification of the functional meaning of ASWA. The neurodevelopmental perspective of psychiatric disorders like schizophrenia [52] suggests that ASWA is a manifestation of disrupted formation of neural circuits, facilitating lasting histological anomalies like gray matter loss, 'disconnection' or the vulnerability for its development. Finally, if 'dysfunctional' implies a transient nature of the phenomenon and not a neurodevelopmentally determined lasting nature, then it should be possible to see changes in ASWA following treatment for the disorder. Given that systematic changes in the intensity of ASWA could be achieved by training in aphasic patients [21] we have started to examine ASWA in patients suffering from depression and posttraumatic stress disorder before and after treatment. Taken together, this might open an intriguing possibility to use ASWA mapping as an additional tool in the diagnostic and prognostic process and in treatment evaluation.

## Abbreviations

ASWA, Abnormal slow wave activity; BDI, Beck Depression Inventory; BPRS, Brief Psychiatric Rating Scale; EEG, Electroencephalography; ICD, International Classification of Disease System; MEG, Magnetoencephalography; MDD, Major Depressive Disorders; MSI, Magnetic Source Imaging; PANSS, Positive and Negative Symptoms Scale; SD, Standard deviation; SSRI, Selective serotonine reuptake inhibitor; TCA, tricyclic antodepressant

## Competing interests

The author(s) declare that they have no competing interests.

## Authors' contributions

BR, CW and TE designed the study and supervised data collection and preprocessing. CW and TE developed and CW implemented the methods of analyses, BR was responsible for recruitment and diagnosis of patients, and accomplished statistical analyses and all authors participated in writing the article, WJR advised data analyses and prepared the paper together with BR and TE.

## Pre-publication history

The pre-publication history for this paper can be accessed here:


